# The Association between Selected Dietary Minerals and Mastitis in Dairy Cows—A Review

**DOI:** 10.3390/ani11082330

**Published:** 2021-08-07

**Authors:** Kacper Libera, Kacper Konieczny, Katarzyna Witkowska, Katarzyna Żurek, Małgorzata Szumacher-Strabel, Adam Cieslak, Sebastian Smulski

**Affiliations:** 1 Department of Preclinical Sciences and Infection Diseases, Poznan University of Life Sciences, Wołyńska 35, 60-637 Poznań, Poland; kacper.libera@up.poznan.pl (K.L.); witko.katarzyna3@gmail.com (K.W.); kacha.zurek@gmail.com (K.Ż.); 2 Department of Internal Diseases and Diagnostics, Poznan University of Life Sciences, Wołyńska 35, 60-637 Poznań, Poland; kacper.konieczny@up.poznan.pl; 3 Department of Animal Nutrition, Poznan University of Life Sciences, Wołyńska 33, 60-637 Poznań, Poland; malgorzata.szumacher@up.poznan.pl (M.S.-S.); adam.cieslak@up.poznan.pl (A.C.)

**Keywords:** mastitis, minerals, immunosuppression, dairy cows, dietary deficiencies

## Abstract

**Simple Summary:**

Inflammation of the mammary gland (mastitis) is an important disease in dairy cows. Among factors affecting the incidence of mastitis, mineral deficiencies are mentioned, since they strongly influence the immune system. Consequently, these deficiencies result in weakened immunity, which increases the risk of any infectious disease. The reviewed minerals (calcium, phosphorus, magnesium, selenium, copper and zinc) interact differently with the immune system; nevertheless, their deficiencies invariably increase the risk of mastitis occurrence in dairy cows.

**Abstract:**

The aim of this paper is to describe the association between selected dietary minerals and mastitis in dairy cows. Minerals are a group of nutrients with a proven effect on production and reproductive performance. They also strongly affect immune system function. In particular their deficiencies may result in immunosuppression, which is a predisposing factor for udder inflammation occurrence. The role of selected dietary minerals (including calcium, phosphorus, magnesium, selenium, copper and zinc) has been reviewed. Generally, minerals form structural parts of the body; as cofactors of various enzymes they are involved in nerve signaling, muscle contraction and proper keratosis. Their deficiencies lead to reduced activity of immune cells or malfunction of teat innate defense mechanisms, which in turn promote the development of mastitis. Special attention was also paid to minerals applied as nanoparticles, which in the future may turn out to be an effective tool against animal diseases, including mastitis. To conclude, minerals are an important group of nutrients, which should be taken into account on dairy farms when aiming to achieve high udder health status.

## 1. Introduction

Mastitis is considered one of the most costly diseases in dairy cows, causing severe losses in the dairy industry [1]. Losses do not only refer to economic issues including milk quality and quantity, antibiotic usage or extra labor; they also refer to the disease significantly affecting animal welfare and public health. The etiology of udder inflammation is mainly associated with bacteria, i.e., staphylococci and streptococci, although viruses, fungi and algae can also cause mastitis [2]. Moreover, non-infectious factors such as genotype, environmental conditions, feed composition and dietary supplement addition may also have an impact on mastitis occurrence and its severity [3]. It is well proven that even in the presence of bacteria the immune system can cope with microbial invasion and prevent the development of inflammation. Any nutrition deficiency will result in a weakened immune response and thus be a predisposing factor for udder inflammation. Minerals are a group of nutrients that has been reported to influence udder health status. Basically, they take part in the formation of structural components of the body and proper functioning of enzymes, hormones, vitamins and cells. By the mid- to late 19th century, it was already known that animals need to consume certain minerals to live and be productive, but their specific role and daily requirements were not recognized [4]. Minerals can be divided into two groups based on their concentration in the organism: macrominerals that are present in the animal body in relatively high concentrations, and trace minerals or microelements found in relatively small amounts in the organism. Macrominerals include calcium (Ca), phosphorus (P), sodium (Na), chlorine (Cl), sulfur (S) and magnesium (Mg), while microelements include iron (Fe), copper (Cu), manganese (Mn), zinc (Zn), cobalt (Co), chromium (Cr), iodine (I), molybdenum (Mb) and selenium (Se) [5].

Microelements occur in relatively small amounts in living organisms. Moreover, aluminum (Al), arsenic (As), nickel (Ni), silicon (Si), tin (Sn) and vanadium (V) are also considered trace minerals, but they are present in extremely low concentrations and their specific roles are not yet fully understood. The reference values of selected serum mineral concentrations in dairy cows are presented in Table 1. The given values collected from selected publications indicate that the production, health and reproduction performance of studied cows was kept at the optimum level, since both deficiencies and excess of dietary minerals can have a detrimental effect on animals, the environment and dairy farm profitability.

In cattle veterinary medicine, mineral deficiencies are mainly associated with characteristic metabolic disorders such as periparturient hypocalcemia (milk fever), hypophosphatemia and hypomagnesemia (grass staggers). However, it has to be remembered that every mineral deficiency leads to immunosuppression [4], which is a well-recognized predisposing factor for the occurrence of infectious diseases including mastitis. Obviously, the key factor determining the concentration of a specific mineral in the body is its supply via feed. Thus, dietary requirements for dairy cows depending of their physiological state are presented in Table 2.

In dairy production, mineral supplementation is a well-proven practice to increase reproductive performance [11,12], but its effect on cow health status including mastitis has also been investigated [13,14]. However, to the best of our knowledge there are practically no papers reviewing in detail the association between minerals and bovine mastitis.

The following part of the text will present selected minerals and the contribution of their deficiencies to immune system dysfunction and consequently the occurrence of udder inflammation.

## 2. Calcium

Calcium (Ca) takes part in various functions in the organism; it forms the structural components of the body and is essential for muscle contraction both in skeletal and smooth muscles including the teat sphincter, in which efficient contraction after milking is crucial to prevent microbial invasion into the udder [15]. In terms of hypocalcemia, contraction of the teat sphincter is impaired [16] and leads to an increased risk of mastitis in Holstein cows. Ca is also considered a blood clotting agent, thus hypocalcemia may result in a reddish coloration of milk due to microhemorrhages in the mammary glands of cows of different dairy breeds [17]. This mineral is also involved in conduction of nerve signals. Cows are relatively susceptible to hypocalcemia due to great losses of this mineral in milk. The crucial period for calcium deficiency to occur is the time within 72 h after parturition. In that period, cows promptly start to produce large amounts of milk and thus excrete considerable amounts of Ca, which may lead to its deficiency [17]. The clinical form of hypocalcemia, known as milk fever or parturient paresis, is a metabolic disorder characterized by lowered serum calcium concentration, usually below 1.5 mmol/L, and specific clinical signs, including prolonged sternal recumbency, loss of appetite, muscle weakness, tachycardia and hypothermia [17]. In extreme cases coma can occur and circulatory collapse may lead to death.

Kimura et al. [18] stated that in Jersey cows before parturition Ca concentration decreases in peripheral blood mononuclear cells leading to the development of hypocalcemia, which contributes to immunosuppression, because calcium is crucial for activating immune system cells [19], in particular neutrophils [20]. It is consistent with the results of studies conducted by Ducusin et al. [21] and Martinez et al. [22], who observed decreased phagocytic activity of neutrophils derived from hypocalcaemic Holstein cows compared to normocalcaemic cows. Those authors indicated subclinical hypocalcemia as a factor predisposing to infections. In a study conducted on Holstein cows by Hisaeda et al. [23], the blood calcium concentration was significantly lower in cows with peracute coliform mastitis compared to healthy cows. Those authors suggested that this phenomenon occurred because of the production of inflammatory cytokines in mastitic cows, which decreases PTH secretion and the conversion of 25-OH vitamin D to 1.25-D and consequently results in a reduced calcium concentration. Meglia et al. [24] found a decrease in concentrations of several minerals, including calcium, in the period around calving in Swedish dairy cows, which was associated with a lower proportion of neutrophils expressing adhesive molecules (CD62L). However, Rodriguez et al. [25] as well as Chamberlin et al. [26] found no statistically significant differences in somatic cell counts between Holstein cows with hypocalcemia and cows with appropriate Ca concentrations.

## 3. Phosphorus

Phosphorus (P), of which 85% is present in the skeletal system, is an essential component of nucleic acids (DNA and RNA) and is contained in high-energy compounds such as ATP [27]. This mineral is also involved in buffering the pH (phosphate buffer) of body fluids. Phosphorus deficiency, especially around calving and in early lactation, has been associated with hampered productivity, feed intake depression and an increased risk of morbidity in fresh cows [27]. When investigating the immune system and resistance to infection, Eisenberg et al. [28] stated that hypophosphatemia negatively affects both phagocytic activity and granulocyte count in transition Holstein dairy cows. However, in research conducted earlier by the same authors [29], granulocytes derived from cows fed a phosphorus-deficient diet were less viable, but phagocytic activity was not affected. Mullarky et al. [30] observed no effect of different P dietary supply levels on the phagocytic activity of granulocytes or lymphocyte proliferation. The mechanism associated with impaired activity of immune cells in cows with phosphorus deficiency has not been fully investigated. Reports on other animal species such as rats suggest that ATP content of leukocytes tends to be lower in hypophosphatemic individuals and that explains their decreased phagocytic activity and proliferation [31]. It is important to note that there are concerns regarding environmental pollution with P of fecal origin, thus it has been suggested that the P content of manure should be limited, thereby enforcing a more restrictive use of P in bovine nutrition [28].

## 4. Magnesium

Magnesium (Mg) plays an essential role in cell metabolism and acts as a cofactor for over 300 enzymes including alkaline phosphatase, phosphomonoesterase, pyrophosphatase and glycolytic enzymes such as hexokinase, phosphoglucomutase, phosphofructokinase, phosphoglycerate kinase, phosphoglycerate mutase and enzymes involved in the citric acid cycle (tricarboxylic acid cycle; Krebs cycle) such as the pyruvate dehydrogenase complex or isocitrate dehydrogenase [32]. In addition, an appropriate intracellular magnesium concentration is required for Na⁺/K⁺-ATPase functioning [32]. The primary site of Mg2+ absorption is the rumen and it may be influenced by diet type and forage type [33]. Referring to the immune system, Mg is an integral part of the innate immune response, id est, the complement system as well as properdin. Generally, to the best of our knowledge, there are no papers regarding magnesium and its role in the bovine immune system; however, many studies have been carried out on rodents. For example, Weglicki et al. [34] observed increased levels of proinflammatory cytokines (IL-6, TNF-α) in rats under Mg deprivation for three weeks. This is in agreement with the findings reported by Malpuech-Brugère et al. [35], who also noticed an increased IL-6 concentration in Mg-depleted rats. Furthermore, Van Orden et al. [36] recorded increased total leukocyte counts in rats fed a diet that was extremely low in Mg compared to the control group (30 ppm). This corresponds with the results of a study performed by Bussiere et al. [37], who noticed an elevated level of IL-6 and an increased count of polymorphonuclear cells in Mg-deficient rats compared to rats in the control. The authors claim that an enhanced inflammatory response is a consequence of Mg deficiency and that the reduced extracellular Mg level might be responsible for the activated state of immune cells. However, there are reports suggesting an association between Mg deficiency and increased prevalence of lameness in cattle [38], or claims that Mg has a promoting effect on both pre-implantation blastocysts and term development post-embryo-transfer in Holstein cattle [39]. Taking into account all these results, magnesium is an important factor for the immune system, but it is not clear if Mg deficiency itself acts as a proinflammatory factor or rather results in immunosuppression, which in turn promotes inflammation. Therefore, further studies of cattle are required to fully understand its role.

## 5. Selenium

Selenium (Se) is a semimetal, which is found in relatively small amounts in animal organisms. In dairy science it is well documented that selenium supplementation can enhance growth, reproductive performance and health status in cattle [40]. Selenium deficiencies may result in calf growth retardation, immunosuppression and difficulties in reproduction. It is involved in antioxidant defense, redox state regulation and a variety of specific metabolic pathways, but some biological functions of selenium are still unknown. It is also a constitutive part of the 21st amino acid named selenocysteine [40].

Referring to the immune system, Se is contained in the active center of the enzyme glutathione peroxidase (GSH-Px), which reduces reactive oxygen species and so has an antioxidant effect. Thus, selenium supplementation may result in positive clinical responses under various conditions with increased oxidative damage such as mastitis [8]. Moreover, studies report that selenium concentration may influence bovine neutrophil performance and their ability to neutralize microorganisms. Neutrophils are considered to be immunocytes, which play an important role during phagocytosis and act in the line of defense against microorganisms invading the mammary gland. In vitro studies based on bovine neutrophils showed that Se supplementation enhances chemotactic migration, phagocytosis and serum superoxide dismutase (SOD) activity [41], and intracellular antibacterial activity against *S. aureus* [42]. More recent studies have suggested [43] that selenium may play a crucial role in immune and inflammatory regulation by influencing the differential expression of exosomal mRNAs of key genes in bovine mastitis. They demonstrated that selenium affects mRNA expression in MAC-T-cell-derived exosomes related to immunity and inflammatory pathways. A study carried out by Wang et al. [44] showed that selenium down-regulates inflammatory mediators TNF-α and IL-1β, and IL-6 gene expression, which may be responsible for the anti-inflammatory effect of Se. In turn, Boyne et al. [45] found neutrophils that were less able to kill *Candida albicans* when they were derived from Se-deficient steers. The above-mentioned in vitro studies have been supported by many field trials. Researchers have reported the highest treatment efficiency when an antibiotic was combined with selenium preparation [46,47] and reported a lower incidence of clinical mastitis when Holstein cows were supplemented with this mineral [48], or a shorter duration of clinical symptoms [49]. The somatic cell count is negatively correlated with the serum selenium level [50]. Selenium significantly inhibits the expression of NLRP3, ASC, Caspase-1, Caspase-1 p20 and Pro-IL-1β. Dietary selenium can attenuate *Staphylococcus aureus* mastitis by the inhibition of the NLRP3 inflammasome [43]. In addition, a beneficial effect on mastitis treatment was observed when affected cows were injected with a multimineral preparation containing Se [41,51]. In contrast, Ganda et al. [13] reported no beneficial effect on overall cure rate of subclinical mastitis when lactating cows were supplemented with a mix of trace minerals. Special attention should be paid to the chemical form of selenium, since for several years scientists have been working on new, less toxic forms of selenium supplementation. Żarczyńska et al. [52] indicated that selenite triglycerides are safe and effective selenium supplements for cattle. Moreover, organic sources of Se (Se yeast, Se-Met, and HMSeBA) are considered efficient bioavailable sources compared with inorganic Se. Nowadays, nano-Se is receiving more attention due to its multiple health benefits compared with inorganic and organic Se sources for use in dairy animals [53]. Gopi et al. [54] indicated that the comparative advantage and efficacy of nanoparticles stem from their smaller particle size and larger surface area, enhanced mucosal permeability, and higher intestinal absorption as a result of nanoemulsion formation. In addition to its enhanced bioavailability, nano-Se also exhibits reduced toxicity and lower antagonism with other minerals compared with other sources of Se. Wichtel et al. [55] stated that measuring selenium concentration in bulk tank milk could be a useful tool to assess herd selenium status. If the concentration is below 0.12 µmol/L of bulk tank milk, selenium deficiency is suspected, while selenium content over 0.28 µmol/L is adequate [55].

## 6. Copper

Copper (Cu) is considered necessary for structural and catalytic properties of cuproenzymes (in Latin cuprum means copper) [56]. These include, inter alia, cytochrome-c oxidase, superoxide dismutase, catechol oxidase, ceruloplasmin and amine oxidases. It is the second most common metal, immediately after zinc, in various enzymes, for which Cu is essential for their appropriate function [57]. It is also involved in collagen and elastin synthesis as well as myelination and hemoglobin production [56].

Considering the immune system and mastitis microorganisms, copper is believed to exhibit antibacterial properties against bacteria isolated from mastitic cows. According to Reyes-Jara et al. [58], Cu concentration as low as 250 ppm inhibits the growth of common mastitis microorganisms such as *Escherichia coli* and coagulase negative Staphylococci. This is consistent with the results reported both by Wernicki et al. [59] and Kalińska et al. [60], who stated that nanoparticles of silver and copper exhibit the highest antimicrobial activity against bacteria isolated from inflamed udders. This suggests that a copper preparation may be considered a reliable alternative to dipping solutions. Furthermore, in vivo studies reported that 100-day dietary Cu supplementation (Cu concentration in the experimental group at 20 ppm vs 6.5 ppm in the control) resulted in a reduced clinical response when Holstein cows were experimentally intramammary infected with E. coli [61]. This is supported by a study carried out by Gakhar et al. [62], who observed a reduced incidence of postpartum mastitis in cows supplemented with copper compared to the non-supplemented group. Copper deficiencies result in impaired phagocytosis and decreased Cu,Zn-SOD activity [63], whereas Cu antimicrobial properties are explained by the disruption of bacterial lipids, proteins and DNA through oxidation [64].

## 7. Zinc

Zinc (Zn) is a microelement that plays a crucial role in rumen microbiota maintenance and synthesis of proteins including collagen, glucagon, insulin, as well as in DNA and RNA synthesis [65]. Zn is an essential activator for the highest number of enzymes including alkaline phosphatase, carbonic anhydrase, DNA and RNA polymerase and (together with Cu) superoxide dismutase, which plays a key role in antioxidant processes [57]. Zn is also a cofactor for a series of oxidoreductases and takes part in keratin formation. In terms of the effect of Zn on health performance in dairy cattle, some studies reported that dietary supplementation of zinc decreased somatic cell count [66,67] and milk amyloid A levels [66]. In contrast, Whitaker et al. [68] recorded no effect of dietary zinc supplementation on somatic cell count. Intact mammary epithelium impenetrable for microorganisms is considered an innate part of the udder immune system. Weng et al. [69] reported improved integrity of mammary epithelium when Holstein cows were supplemented with Zn preparations; however, conflicting results were obtained by Shaffer et al. [70]. Zinc is crucial for the development and proper function of cells mediating innate immunity such as neutrophils. Deficiency of this mineral adversely affects the growth and function of T and B cells. Zn exhibits antioxidant properties and stabilizes membranes, which suggest its role in the prevention of free-radical-induced injury during inflammatory processes [71].

## 8. Mineral Nanoparticles—A Promising Tool in Udder Inflammation Management

In animal production, minerals can be incorporated into the diet or used in therapy in different forms, including inorganic salts, organic forms, chelates or as nanoparticles. Many reports have shown evidence that nanoparticles may be good candidates for animal growth promoters, antimicrobials and promising future alternatives to conventional cleaning agents [72]. Nanoparticles (NPs) including nanometals and nanometal oxides are defined as nanosized structures with one or more of their dimensions (length, width or thickness) in the nanometer range of 1–100 nm [72]. The most important advantage of NPs is that they do not lead to bacterial resistance [73]. Using them as a new dipping solution is currently considered. NPs may have a toxic effect on bacteria because of the formation of reactive oxygen species (in the Fenton reaction), DNA degradation, and lipid and protein peroxidation [74]. In the literature, nanoparticles containing copper (CuNPs), silver (AgNPs), platinum (Pt_NPs) and zinc (ZnONPs) have been described [60]. For example, it has been reported that CuNPs demonstrate significant inhibitory activity against several bacteria species, e.g., *Escherichia coli*, *Klebsiella pneumoniae*, *Pseudomonas aeruginosa*, *Propionibacterium acnes* and *Salmonella typhi*. Moreover, CuNPs display an antifungal activity against the *Candida* species [75], which can cause mastitis. Furthermore, *S. aureus* strains isolated from clinical and subclinical cases of mastitis exhibit significant susceptibility to AgNPs and AuNPs [76]. In addition, ZnO-NPs also exhibit antimicrobial properties against *S. aureus* and other pathogenic bacteria such as *E. coli* and *K. pneumoniae* [77]. Previous studies showed that AgNPs can be used in diseases caused by algae [78] that are also involved in udder inflammation. According to Wernicki et al. [59], AgNPs and CuNPs may be the most effective solution in mastitis management due to their synergistic effect on various pathogens. An in vitro study revealed that the oxidoreductive activity of BME-UV1 cells increased after incubation in media containing 0.5 and 1 mg/mL of AgNPs and in media containing 0.1, 0.5 and 1 mg/L of AgCuNPs. However, higher concentrations of selected NPs decreased the oxidoreductive activity of BME-UV1 cells. Conversely, data on HMEC cell viability after incubation in media containing AgNPs, CuNPs or AgCuNPs at concentrations of 0.1, 0.5, 1, 2 and 2.5 mg/L showed no decrease in the oxidoreductive activity of HMEC cells and therefore they did not have any cytotoxic effect [60]. However, a question arises concerning the safety of NP administration. It is important to highlight that limited in vivo evidence is available to support many applications demonstrated in vitro [72]. The reports are not consistent. On the one hand, silver nanoparticles are reported to be cytotoxic and genotoxic for human microvascular endothelial cells [79]. On the other hand, histopathological examination of various organs from treated rats shows that AgNPs and AuNPs are not toxic at low doses [76]. Furthermore, the NPs analyzed in this study did not reveal a toxic effect on BME-UV1 or HMEC cells, but an elevated LDH level was observed after the incubation of NPs with BME-UV1 cells. Nevertheless, the dose and form of applied NPs seem to be key factors determining toxicity. Therefore, the addition of NPs should potentially be safe for cattle and humans, but further studies are required.

## 9. Mineral Supplementation as an Auxiliary Tool in Mastitis Treatment—Field Trials

From a clinical point of view, it is well-known that some mastitis cases require supportive therapy including fluids with calcium, since cows with udder inflammation are often hypocalcaemic [80]. Besides, the injectable supplementation of trace minerals, such as zinc, manganese, selenium and copper, can also be considered as an auxiliary tool in therapy of mastitis in dairy cows. For example, Hoque et al. [47] claimed that antimicrobial therapy is the most effective in mastitis treatment; however, in the same experiment cows injected only with selenium preparations were less prone to udder inflammation compared to the untreated control group. Ganda et al. [13] demonstrated that injection of trace minerals (including zinc, manganese, selenium and copper) reduces the number of chronic mastitis cases, but has no impact on the incidence of clinical cases requiring treatment. Machado et al. [14] showed that injection of a multimineral preparation (including selenium, copper, zinc and manganese) had a positive impact on udder health, decreasing linear somatic cell count (SCC) scores and the incidence of subclinical and clinical mastitis. Moreover, the same researchers reported that administration of this multimineral preparation increases serum superoxide dismutase (SOD) activity, but does not subsequently affect leukocyte function [41]. However, Ferreira and Petzer [81] observed no evident correlation between SCC and the milk or serum selenium values in cows supplemented with Se in different forms, while in a study performed by Bourne et al. [82], supplementation of vitamin E with Se reduced the risk of culling and mastitis rate by 10 %. Recently, Smulski et al. [83] confirmed that administration of an antibiotic combined with antioxidant including selenium slightly improves the effectiveness of clinical mastitis treatment.

**Figure 1:**
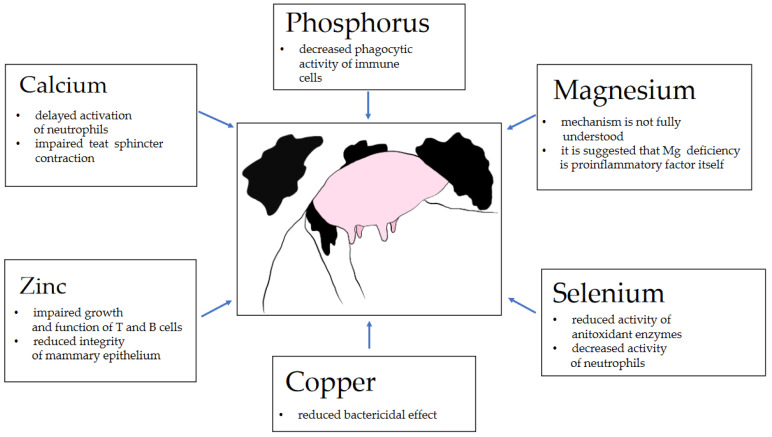
Brief summary of pathomechanisms related to mineral deficiencies and mastitis incidence.

To recap, a brief summary of the pathomechanisms related to mineral deficiencies and mastitis incidence is given in Figure 1.

## 10. Conclusions

When managing nutrition on a dairy farm, special attention should be paid to minerals because they are involved in various biological processes in cows and hence influence key traits in dairy production. Moreover, minerals are essential for the proper function of immune cells, so any mineral deficiency may lead to suppression of immunity, which predisposes cows to infection. Mastitis is an ongoing problem even on well-managed farms, and mineral supplementation might be a way to enhance the innate immunity of the mammary gland and thus contribute to a decreased risk of udder inflammation.

## Figures and Tables

**Table 1:** Reference values of serum mineral concentrations in dairy cows.

Mineral	Serum Concentration	Reference
Calcium (Ca)	2.2–2.6 mmol/L	[6]
Phosphorus (P)	1.3–2.6 mmol/L	[6]
Magnesium (Mg)	0.75–1.0 mmol/L	[7]
Selenium (Se)	0.73–1.08 µmol/L	[8]
Copper (Cu)	1–18 µmol/L	[9]
Zinc (Zn)	8–19 µmol/L	[10]

**Table 2:** Daily dietary requirements for selected minerals in dairy cows taking into account their physiological state (lactating/non-lactating) according to the Nutrient Requirements of Dairy Cattle [5].

Mineral	Requirement for Non-Lactating Cows	Concentration in Milk (mg/kg)	Requirement for Lactating Cows
Calcium (Ca)	0.0154 g/kg BW	1220	0.106 g/kg BW
Phosphorus (P)	1 g/kg DMI	900	2.5 g/kg DMI
Magnesium (Mg)	3 mg/kg BW	150	10 mg/kg BW
Copper (Cu)	152 mg/cow (15.2 mg/kg DMI)	0.015	313 mg/cow (15.7 mg/kg DMI)
Selenium (Se)	0.3 mg/kg DMI	0.01–0.025	0.3 mg/kg DMI
Zinc (Zn)	310 mg/day (31 mg/kg DMI)	4	1261 mg/day (63 mg/kg DMI)

Assumptions: 650 kg body weight (BW) Holstein cow, 40 kg of milk per day if lactating, dry matter intake (DMI) in lactating/non-lactating cow equals 20/10 kg per day.

## Data Availability

Not applicable.
